# Coherence, not conditional meaning, accounts for the relevance effect

**DOI:** 10.3389/fpsyg.2023.1150550

**Published:** 2023-05-15

**Authors:** Maxime Bourlier, Baptiste Jacquet, Daniel Lassiter, Jean Baratgin

**Affiliations:** ^1^Université Paris 8, Laboratoire Cognition Humaine et Artificielle (CHArt, RNSR 200515259U), Saint-Denis, France; ^2^Probability, Assessment, Reasoning and Inferences Studies Association, Paris, France; ^3^School of Philosophy, Psychology and Language Sciences, University of Edinburgh, Edinburgh, United Kingdom

**Keywords:** conditionals, discourse coherence theory, hypothetical inferential theory, pragmatics, relevance effect

## Abstract

Missing-link conditionals like “If bats have wings, Paris is in France” are generally felt to be unacceptable even though both clauses are true. According to the *Hypothetical Inferential Theory*, this is explained by a conventional requirement of an inferential connection between conditional clauses. Bayesian theorists have denied the need for such a requirement, appealing instead to a requirement of discourse coherence that extends to all ways of connecting clauses. Our experiment compared conditionals (“*If A, C*”), conjunctions (“*A and C*”), and bare juxtapositions (“*A. C*.”). With one systematic exception that is predicted by prior work in coherence theory, the presence or absence of an inferential link affected conditionals and other statement types in the same way. This is as expected according to the Bayesian approach together with a general theory of discourse coherence.

## 1. Introduction

The call by Oaksford and Chater (2001, 2007) for a paradigm shift in the psychological study of reasoning to take into account the probabilistic nature of reasoning has undergone major development in recent years and has provided many explanations for the inferences and representations of logical arguments implemented in everyday life (for comments: Over, 2009, 2020, 2021; Evans, 2012; Elqayam and Over, 2013; Oaksford and Chater, 2013; Baratgin et al., 2015; Johnson-Laird et al., 2015; Baratgin and Politzer, 2016; Elqayam, 2017; Knauff et al., 2021; Oaksford, 2021; Cruz, 2022; Douven, 2022; Johnson-Laird and Khemlani, 2022). This new paradigm gathers two families of theories that make use of Bayesian concepts in somewhat different ways (Elqayam and Evans, 2013; Douven et al., 2022). “Strict Bayesians” tend to lean closer toward classical Bayesian precepts, explaining deviations from these norms in terms of interactions with other systems—for example, conversational pragmatics and details of natural language semantics (Baratgin, 2002; Baratgin and Politzer, 2006; Cruz et al., 2016; Lassiter and Baratgin, 2021; Cruz and Over, 2023; Over and Cruz, 2023). “Soft Bayesians”, for instance proponents of the *Hypothetical Inferential Theory* (HIT) agree on the importance of uncertainty and subjective degrees of belief in reasoning but reject some aspects of Bayesian orthodoxy (Douven and Verbrugge, 2012; Douven, 2015, 2017, 2022; Krzyżanowska et al., 2017, 2021; Douven et al., 2018, 2022; Krzyżanowska and Douven, 2018; Mirabile and Douven, 2020).

These perspectives have been reflected in recent debates about the semantics and pragmatics of indicative conditionals (for a recent discussion Oaksford and Chater, 2020; Berto, 2022; Sikorski, 2022). Both sides take as a starting point the suggestion of Ramsey (1990) that we evaluate “If A, C” by supposing that A is true.^1^ Strict Bayesians adopt “Stalnaker's Thesis” or *The Equation*, according to which one's degree of belief in “If A, C” should agree with her assessment of *P*(*C*∣*A*) (Stalnaker, 1970; Evans and Over, 2004; Wang et al., 2022). They argue in addition for the “Strong Centering” principle, in which the truth of both “A” and “C” is sufficient to ensure the truth of “If A, C” (Cruz et al., 2016).^2^

Conditionals with true antecedents and consequents are generally accepted in experiments (Cruz et al., 2015; Politzer and Baratgin, 2016; Shao et al., 2022), as Strong Centering would lead us to expect. However, “missing-link” conditionals—those in which the two clauses are unrelated to each other—are an important exception. Even when the antecedent and consequent are clearly true, participants do not consistently endorse conditionals like those in (1) (Matalon, 1962; Skovgaard-Olsen et al., 2017; Vidal and Baratgin, 2017; Krzyżanowska and Douven, 2018).

(1) a. If elephants are gray, then 2+2 = 4 (Matalon, 1962, p. 82).
b. If Napoleon is dead, Oxford is in England (Edgington, 1995, p. 268).

This apparent need for an inferential link from the antecedent to the consequent in order for a conditional to be accepted as true is called the *relevance effect* (Skovgaard-Olsen et al., 2016).

HIT explains the *relevance effect* by positing that “If A, C” is true only when “*C*” follows from “*A*” by some form of (deductive or non-deductive) inference. Crucially, this means rejecting Strong Centering, since the truth of “A and C” does not guarantee an inferential connection between them. HIT also rejects Stalnaker's Thesis: one can have high credence in “A and C” and yet be certain that the conditional is false due to lack of an inferential connection.

In response, strict Bayesians have offered a pragmatic explanation of the relevance effect related to the notion of *coherence*, as already anticipated by Matalon (1962).^3^ This idea was explored more deeply by Ducrot (2008). According to Ducrot, the primary aim of a conditional *if A, C* is not to signify an inferential linking between “A” and “C”, but to perform a complex illocutionary act consisting of two acts in succession: to (a) imagine “A”, and then (b) affirm “C”. A speaker's choice to suppose “A” before affirming “C” suggests that there is some kind of connection between “A” and “C”. Otherwise, we would not be able to rationalize why the speaker would precede the act of affirmation by an act of supposition.

Following this reasoning, Cruz et al. (2016) provided evidence that two utterances in any type of statement would require a “common topic of discourse” in order to be acceptable. HIT theorists have responded with several studies arguing that the presence of a common topic is not sufficient to explain the relevance effect (Krzyżanowska et al., 2017, 2021; Skovgaard-Olsen et al., 2019; Rostworowski et al., 2021). Recently, Lassiter (2022) proposed a new account drawing from the theory of *discourse coherence* (Kehler, 2002).

## 2. Discourse coherence and the relevance effect

*Discourse Coherence Theory* (hereafter DCT) attempts to account for the way we connect the informational contribution of different clauses in a discourse into a coherent whole (Hobbs, 1979; Knott and Dale, 1994; Kehler, 2002, 2006; Asher and Lascarides, 2003; Wolf et al., 2004). For instance, when reading (2) below we naturally infer that John is taking the train in order to visit his family, even though there is nothing in the literal content of the text to indicate this.

(2) John took the train from Paris to Istanbul. He has family there (Kehler, 2006, p. 2).

DCT shows that we supply additional information, going beyond the literal content of the text, as a byproduct of an obligatory process of understanding sequences of utterances as coherent texts. We tend to enrich (2) with the information that the clauses are in an *Explanation* relation: visiting family is the reason for John's trip.

Kehler (2002) describes a variety of coherence relations that can hold between clauses. Kehler's *cause-effect relations*— (a) *result*, (b) *explanation*, (c) *violated expectation*, and (d) *denial of preventer* —are of particular interest to us in this study.^4^

As Lassiter (2022) discusses, the first two relations—result and explanation—indicate informational relevance between the two clauses: the first in some sense explains the second, or vice versa. We will group these together as *inferential readings*.

(3a) If Lucy is tall enough, she will be able to ride the carousel.
(3b) Lucy is tall enough. She will be able to ride the carousel.

(4a) If the vase is broken, someone dropped it.
(4b) The vase is broken. Someone dropped it.

These *inferential readings* match the definition of an *inferential link* proposed for conditionals by HIT theorists. However, Lassiter argues that the inferential link present in (3a) and (4a) does not come from the semantics of conditionals. Rather, it is explained by DCT, just like in the non-conditional examples (3b) and (4b).

In contrast, the *violated expectation* and *denial of preventer* relations indicate a sort of independence: the event in the second clause happened *even though* the information in the first clause would have led us to expect otherwise, or vice versa. Cruz and Over (2023) have called conditionals containing such relations *independence conditionals*, therefore we will group these relations together as *independence readings*. Independence readings appear in conditionals, as in other types of texts.

(5a) If you press the power button, the TV does not turn on.
(5b) John pressed the power button. The TV did not turn on.

(6a) If Martin arrived on time, his bus still left without him.
(6b) Martin arrived on time. His bus still left without him.

Both examples convey that the second clause would have been true *whether or not* the first clause was, and that the resulting lack of connection between clauses is surprising given background knowledge (see Lassiter 2022: Section 4.4). For instance, (5a) indicates that pressing the button has *no effect* on the state of the television (although one would normally have expected otherwise). This is also the case in (5b), suggesting again that conditionals behave similar to other ways of connecting clauses. As a result, these examples do not fit the basic prediction of *HIT*, which requires that the antecedent's truth should make it possible to infer the truth of the consequent.

As it has been developed, *HIT* is limited to making predictions about “standard” conditionals, i.e., those that display an inferential link. This means that we cannot attribute to *HIT* any direct predictions about non-conditional sentences like (5b) and (6b). However, such parallels between conditional and non-conditional sentences call out for an explanation, and *HIT* is forced to treat them as an unexplained coincidence, generated by the behavior of “non-standard” conditionals that are put aside as being outside the scope of the theory. Indeed, *HIT* offers no empirical or theoretical criteria for identifying which examples are “standard” except to inspect them and consider whether they conform to the theory's predictions.

The parallels between conditional and non-conditional sentences just noted suggest instead that the dual behavior of *if*-sentences should receive a unified semantic-pragmatic explanation. *DCT* offers such an account, and it provides a set of semantic and pragmatic diagnostics that distinguish inferential and independence conditionals in terms of background assumptions and the availability of various discourse particles (Lassiter, 2022).

Our core hypothesis is that the relevance effect does not depend on the conditional form itself, but rather on our ability to find a coherent reading between utterances. Therefore, we expect conditionals interpreted with an *inferential* or *independence* reading to maintain this interpretation in other text types, such as conjunction and juxtaposition, as long as the relevant interpretation is compatible with the meaning of the connective device and other aspects of the form (e.g., intonation and discourse particles). The broad prediction of a parallel between conditional and non-conditional connective devices should hold whether or not there is an inferential link between clauses. If this parallel does hold, it would provide support for DCT and a further explanatory challenge for HIT.

## 3. Experiment

### 3.1. Participants

We collected data from 50 participants (33 women, 15 men, two declined to comment). Data from one additional participant was discarded because the participant was not a native French native speaker. The average age was 36.2 years (SD: 15.1, minimum 19, maximum 67). Level of education ranged from no high school degree to postgraduate degree. The participants were recruited via social networks.

### 3.2. Material and procedure

Our questionnaire was designed on the SoSci Survey website. The material was built in French from 8 pairs of statements with different intended coherence relations: Four that we judged to convey relevance (two for *result*, two for *explanation*) and four that we judged to convey the absence of a previously expected relevance relation (two for *denial of preventer*, two for *violated expectation*). Those 8 pairs were then crossed with three connective devices (conditional, conjunction, and juxtaposition of sentences) for a total of 24 items (see Supplementary material). We used a within-subjects design. We therefore had 24 responses per participant, for a total of 400 responses per statement form (conditionals, conjunctions and juxtapositions).

**Figure 1:**
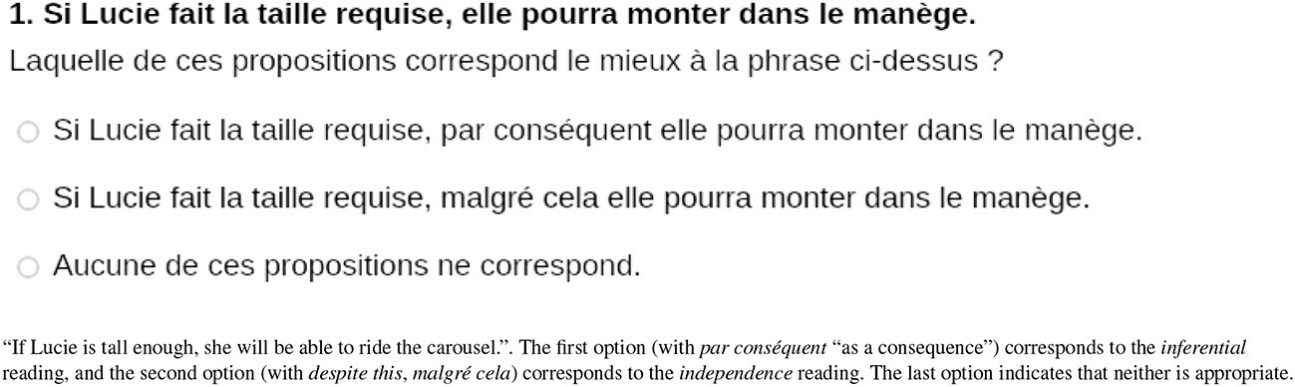
Item example.

Each item was presented on a separate page in random order (Figure 1), with one of the 24 statements followed by three response options. Participants were asked to choose the option that best described their interpretation of the statement above. Two response modalities were variants of the statement. In the first, we added *par conséquent* (“as a result”) to force an *inferential* reading. Note that the French phrase is compatible with both a predictive reading (Result relation) and a diagnostic reading (Explanation relation). The second candidate paraphrase added *malgré cela* (“despite this”) to force an *independence* reading. The third response option indicated that neither of the paraphrases offered corresponded to the participant's interpretation. The participant pressed the *next* button to continue to the next item.

Following this, eight questions were used to verify that the statements had been understood by the participants as either having the intended inferential link or lack thereof. Participants were asked to rate on a 4-point Likert scale to what extent they would be able to conclude element “*C*” from element “*A*” for each of the eight statements.

Finally, one page was devoted to demographic questions (gender, age, level of education, and whether or not they were native French speakers).

### 3.3. Predictions

Broadly speaking, the strict Bayesian-cum-DCT theory predicts that responses should be sensitive to Relation, but independent of Form. When participants assume the presence of an inferential link, this is attributed to their choice to interpret the text using an inferential coherence relation (result or explanation). When an inferential link is implausible, participants can also assign an *independence* reading. When neither option is plausible, the text should be felt to be incoherent. These options apply in the way to any type of text.

However, the prediction of form-independence holds only if all of the clausal connective devices used are semantically compatible with all of the coherence relations naturally associated with the texts. As we will see below, prior research (Txurruka, 2003; Asher and Vieu, 2005) provides independent reason to expect semantic incompatibility between one of our connective devices (conjunction) and a class of coherence relations called *Subordinating*, including our explanation relation. This point motivates the revised analysis in Section 4.1, which tests the hypothesis that coherence is independent of form for non-subordinating coherence relations.

Extracting direct predictions from HIT is somewhat difficult. As noted above, HIT does not address non-conditional sentences, and so in principle the theory makes no predictions at all about our experiment. However, the need for a theory of discourse coherence in language understanding is not controversial, and we assume that HIT theorists would agree that the coherence relations we have discussed exist and are relevant. If so, then the effect of HIT should be, in broad terms, to exclude Independence readings as semantically incompatible with the relevance requirement of “if”. This would have the effect of making participants more likely to endorse Inferential relations more with *if* than with sentence types with which they are compatible.

Admittedly, it is difficult to apply this prediction with precision because HIT also has recourse to a notion of “non-standard” conditionals to which the relevance effect does not apply. In principle, a HIT theorist could argue that any pattern of responses at all is compatible with the theory: responses indicating Irrelevance relations can simply be treated as “non-standard”. However, note that HIT explicitly treats relevance-enforcing (“standard”) readings of conditionals as a default option that is statistically more prominent than Irrelevance readings (e.g., Douven, 2008, p. 31; Douven et al., 2018, p. 54). Given this, HIT leads to the broad prediction that the use of an “if”-sentence should bias participants toward Inferential responses, and should do so to a greater extent than (for example) juxtaposition, which is equally compatible with inferential and independence interpretations.

On this reasoning, we expect that HIT would be supported by response patterns where the choice of a conditional renders an independence reading less likely than it would be with juxtaposition or conjunction.

### 3.4. Data analysis

We computed Bayes factors, BIC ^5^ (Bayesian Information Criterion) and AIC ^6^ (Akaike Information Criterion) values using multinomial mixed models to take into account repeated measures per participant.^7^ The Bayes factor is the ratio of likelihoods of the data given the competing hypotheses:


(1) $$\begin{array}{l} BF_{10}=\frac{P\left(D|H_{1}\right)}{P\left(D|H_{0}\right)} \end{array}$$

One issue with the Bayes Factor is that it is quite complex to calculate. We use the BIC approximation to calculate the Bayes Factor, which is similar to a calculation of the Bayes Factor with a unit prior (Raftery, 1999):


(2) $$\begin{array}{l} BF_{10}\approx e^{\frac{BIC_{1}-BIC_{0}}{2}} \end{array}$$

Following Andraszewicz et al. (2015) (whom themselves adapted a table from Jeffreys, 1961), we consider a Bayes Factor above 3 to show a tendency in favor of the model compared to the alternative model, and a Bayes Factor above 10 to be strong evidence in favor of the model compared to the alternative model. To be conservative, we consider both BIC and AIC, since the former tends to prefer too-simple models while the latter tends to prefer too-complex models (Kuha, 2004).

Table 1 shows the models we considered. Model 0 is the baseline model in which none of the factors has an effect on participants' interpretations. In Model 1, only Form (conditional, conjunction, or juxtaposition) affected responses. According to Model 2, only the presence or absence of an inferential link affected responses. In Models 3 and 4 (Main Effects Link and Full Link) we considered both Form and Link to have an effect, either additional in Model 3 or with an interaction in Model 4. Model 5 considered a main effect of Relation (Result, Explanation, Violated Explanation, or Denial of Preventer). Model 6 (Main Effects Relation) added Form as a main effect, and Model 7 considered an interaction between Relation and Form.

**Table 1:** Summary of the models used in the data analysis for predicting participant's reading.

	**Parameters**	
**Model name**	**Fixed effects**	**Random effect**
Model 0 (Null)	None	Intercept
Model 1 (Form)	Form	Intercept
Model 2 (Link)	Link	Intercept
Model 3 (Main Effects Link)	Form+Link	Intercept
Model 4 (Full Link)	Form*Link	Intercept
Model 5 (Relation)	Relation	Intercept
Model 6 (Main Effects Relation)	Form+Relation	Intercept
Model 7 (Full Relation)	Form*Relation	Intercept

## 4. Results

A summary of the comparisons done between the models can be found in Table 2. Model 6 (Main Effect Relation) was the best model of the data according to the Bayes Factor, and BIC (*BF*_60_ = 1.1*e*+140, *AIC* = 952, *BIC* = 987), followed by model 5 (Relation). Model 7 (Full Relation) fared slightly better than all other models on AIC, which (as noted above) is known to be biased toward complex models (Kuha, 2004). While AIC and BIC metrics were split, the Bayes Factor indicated extreme evidence for Model 6 (*BF*_67_ = 7595) compared to model 7 and anecdotal evidence compared to model 5 (*BF*_65_ = 3). A visualization of the data can be seen in Supplementary material and a summary in Table 3.

**Table 2:** Comparison of the models for answers between *inferential* reading, *independence* reading, and neither: the type of relation and the form of the statement have an effect.

**Model name**	**AIC**	**BIC**	***BF*_*X*0_**	***BF*_6*X*_**	
Model 0 (Null)	1,622	1,632	1	1.1e+140	
Model 1 (Form)	1,618	1,638	22	2.3e+141	
Model 2 (Link)	1,046	1,062	7.5e+123	1.4e+16	
Model 3 (Main effects link)	1,036	1,061	1.0e+124	1.1e+16	
Model 4 (Full link)	1,033	1,068	2.1e+122	5.3e+17	
Model 5 (Relation)	964	989	3.6e+139	3	
Model 6 (Main effects relation)	952	987	1.1e+140	1	⋆
Model 7 (Full relation)	938	1,005	1.4e+136	7595	

⋆ Best model: Participants respond in the same way no matter the form of the statement. Only the presence or absence of the inferential link has an effect.

*BF*_*X*0_: Bayes Factor indicating how the given model (X, from 0 to 7) compares to the null model (0). The greater the number the better the model.

*BF*_6*X*_: Bayes Factor indicating how much better the best model (6) was compared to the given model (X, from 0 to 7). For example, Model 6 is 7595 times as likely as Model 7 to explain our data.

**Table 3:** Number of readings between conditions (*N* = 1, 200, *Participants* = 50).

**Condition**			**Readings**			
**Inferential link**	**Intended relation**	**Statement type**	**Relevance**	**Independence**	**Neither**	**Total**
Present	Result	Conditional	87	0	13	100
		Conjunction	87	2	11	100
		Juxtaposition	82	1	17	100
	Explanation	Conditional	79	0	21	100
		Conjunction	43	11	46	100
		Juxtaposition	57	5	38	100
Absent	Violated expectation	Conditional	16	55	29	100
		Conjunction	19	58	23	100
		Juxtaposition	11	66	23	100
	Denial of preventer	Conditional	4	71	25	100
		Conjunction	1	91	8	100
		Juxtaposition	5	87	8	100

We also verified that our items designed to be interpreted with and without inferential links were perceived as such by participants. We analyzed the responses to the 8 verification items for the presence of an inferential link or not. A paired samples Wilcoxon test (since the responses did not follow a normal distribution) shows a highly significant difference (*W* = 30, 433, *p* < 0.001) between the two types of stimuli.

### 4.1. Revised analysis

In our initial analysis, we noticed that materials intended to evoke an explanation relation behaved differently from others when it was paired with coordination: participants selected independence readings, or rejected both options, far more often than we had expected based on other conditions. On reflection, this pattern is readily intelligible based on existing findings in the literature on coherence relations and their linguistic interactions. As discussed by Asher and Lascarides (2003); Asher and Vieu (2005), coherence relations fall into two classes—“coordinating” and “subordinating”—which are distinguished by a variety of empirical criteria, such as the availability of various types of anaphora. Result is a coordinating relation, while explanation is a subordinating relation.

Crucially, Txurruka (2003) demonstrates that the connective “and” is quite generally incompatible with subordinating discourse relations, but it can be used with a wide variety of coordinating relations. For example, consider the examples below. The coordinating relations Result and Violated Expectation are both compatible with all three connective types we have considered. However, an Explanation relation is not possible in (11c), the variant with *and*.

(7) Result
  a. Bill dropped the vase. It broke.
  b. If Bill dropped the vase, it broke.
  c. Bill dropped the vase and it broke.

(9) Violated Expectation
  a. Bill dropped the vase. Nothing happened.
  b. If Bill dropped the vase, nothing happened.
  c. Bill dropped the vase and nothing happened.

(11) Explanation
  a. The vase broke. Someone dropped it.
  b. If the vase broke, someone dropped it.
  c. The vase broke and someone dropped it.

Example (11c) is the odd one out: it is not possible to interpret this sentence as indicating that the vase broke because someone dropped it. Since no other connection is plausible, the result is an incoherent text. Txurruka (2003) shows that this is example is an instance of a general prohibition on subordinating discourse relations in *and*-sentences. Asher and Vieu (2005) discuss a number of further empirical diagnostics and theoretical applications of the distinction between coordinating and subordinating discourse relations.

For our purposes, the restriction of *and* to coordinating relations implies that participants should have been inclined to reject the intended interpretation of explanation stimuli that we constructed with “and”. Depending on the example, they might have succeeded in imposing a result interpretation, an irrelevance reading, or simply concluded that none of the available readings was appropriate.

While we did not foresee this issue when constructing our experimental materials, we decided that a reasonable *post hoc* analysis would exclude data points that were intended to evoke the problematic explanation relation. With this condition removed, the general predictions described above for DCT and HIT should hold: HIT predicting an interaction between form and relation (as in Model 7), and DCT predicting an effect of relation alone (Model 5).

To this effect, we ran the analyses again, but this time without the explanation relation. The models resulting from this analysis can be found in Table 4.

**Table 4:** *Post-hoc* comparison of the models for answers between *inferential* reading, *independence* reading and neither.

**Model name**	**AIC**	**BIC**	***BF*_*X*0_**	***BF*_5*X*_**	
Model 0 (Null)	1,166	1,175	1	2.9e+126	
Model 1 (Form)	1,169	1,188	0.002	1.7e+129	
Model 2 (Link)	602	616	2.4e+121	1.2e+5	
Model 3 (Main effects link)	603	627	7.3e+118	3.9e+7	
Model 4 (Full link)	608	641	8.6e+115	3.3e+10	
Model 5 (Relation)	574	593	2.9e+126	1	⋆
Model 6 (Main effects relation)	576	604	9.2e+123	311	
Model 7 (Full relation)	578	626	1.9e+119	1.5e+7	

Examples that evoke an explanation relation have been excluded, as explained in the main text. The comparison indicates strong support for a model, with no effect of the form of the statement.

⋆ Best model: Participants respond in the same way no matter the form of the statement. Only the presence or absence of the inferential link has an effect.

*BF*_*X*0_: Bayes Factor indicating how the given model (X, from 0 to 5) compares to the null model (0). The greater the number the better the model.

*BF*_5*X*_: Bayes Factor indicating how much better the best model (5) was compared to the given model (X, from 0 to 7). For example, Model 5 is 311 times as likely as Model 6 to explain our data.

In terms of Bayes Factors, the revised analysis strongly supported Model 5—with no interaction between intended coherence relation and choice of form (conditional, conjunction, or juxtaposition)—over the HIT-friendly Model 7 (BF_57_ = 1.5e+7). Model 5 was also the best model according to both AIC and BIC (574 and 593, respectively, as compared to 578 and 626 for Model 7). Overall, these results suggest that discourse coherence was the primary determinant of our participants' responses. Setting aside the orthogonal interaction between “and” and explanation relations, the choice of conditional or non-conditional form appears to have played little or no role in participants' interpretations.

## 5. Discussion

Our study lends support to an interpretation of the relevance effect in terms of discourse coherence. We compared three ways of combining clauses: conditionals, conjunctions, and juxtapositions. Our prediction was that participants would interpret all three with the same type of reasoning about possible relations of coherence among the constituent clauses, using independently motivated inferential relations (*result* and *explanation*) and independence relations (*violated expectation* and *denial of preventer*). Our initial analysis was inconclusive, because of an unanticipated interaction with a general prohibition on explanation relations with *and*. However, when the problematic condition was excluded from the analysis, we found that a model of participants' preference for independence or inferential readings that did not include information about the statement's form led overall to a better fit to the data, as predicted by the coherence approach.

This result suggests that our participants posited an inferential link between clauses in a similar way whether or not a conditional was involved. This suggests that the need for a link between antecedent and consequent in conditionals can be explained without treating it as a conventional feature of conditional semantics, as posited by HIT. Instead, the presence or absence of a link are explained in terms of general reasoning about whether the most plausible interpretation involves an *inferential* coherence relation or an *independence* relation. This result does not, of course, demonstrate conclusively that conditionals have no special features: conceivably, there could be special features that did not, for some reason, appear in our materials. However, the result does place a strong constraint on future work: arguments for the special status of conditionals must control for coherence relations, in order to avoid the possibility that the effect demonstrated is merely due to discourse coherence.

In some cases, conditionals were rejected, indicating either that (a) participants were unable to identify a plausible coherence relation between the antecedent and the consequent, or (b) the most plausible coherence relation was one that did not appear among our response options (parallel, elaboration, etc.). This result occurred in similar ways in all of our statement types, as expected according to DCT. Our results also confirmed the intuitive claim around (5a) that some missing-link conditionals are acceptable because they can be interpreted with an independence reading.

Because of the theoretical flexibility provided by HIT's distinction between “standard” and “non-standard” conditionals, our result does not—and could not—provide a conclusive refutation of this theory. The challenge to HIT is primarily an explanatory one: given that variation in responses was well-explained by a broader theory that is needed in any case to account for coherence effects, it is not clear why HIT would be needed. In addition, as explained in Section 3.3 above, the most plausible reading of HIT as a “default”, together with general principles of discourse coherence, does generate a predicted interaction that was not evidenced in our results. Our experiment thus contributes a major explanatory challenge to HIT. In contrast, the strict Bayesian theory of conditionals, together with DCT, accounts in a single theory for the acceptability of all types of indicative conditionals.

Our results are in apparent conflict with the experimental findings of Krzyżanowska et al. (2017), who found that participants judged sensible certain dialogues that share a “common topic” but lack an inferential connection, even though matched conditionals were not. However, as Lassiter (2022) notes, there is an important confound in the experimental materials: Some Stimuli used were naturally read using further coherence relations such as Parallel and Contrast, which are available in juxtapositions and dialogues but systematically unavailable in conditionals. In contrast, our materials were limited to coherence relations that are independently established to be possible in all types of clause combinations that we used. This difference may account for the apparent contradiction between our results and those of Krzyżanowska et al. (2017).

## 6. Conclusion

The relevance effect has been taken in recent research to call into question core principles of the strict Bayesian approach to conditionals, particularly Strong Centering and Stalnaker's Thesis. HIT theorists have argued that the need for an inferential connection is a conventional feature of conditionals. In this study, participants interpreted statements involving two true clauses that varied in the presence or absence of an inferential link. Our results showed little or no evidence that the choice of conditional, conjunction, or juxtaposition interacts with the presence or absence of an inferential link. From the perspective of DCT, it appears that statements of all types were interpreted in the same fashion—with an inferential reading, an independence reading, or neither depending on the content of the clauses, as long as the coherence relation was semantically compatible with the connective device. If this is correct, apparent threats to Strong Centering can be explained by a failure of discourse coherence. This result lends support to the suggestion of Matalon (1962) and Cruz et al. (2016), as refined by Lassiter (2022): the relevance effect in conditionals can be attributed to the fact that a listener has difficulty rationalizing why a speaker would choose to connect these clauses in this particular way.

## Data availability statement

The datasets presented in this study can be found in online repositories. The names of the repository/repositories and accession number(s) can be found below: https://doi.org/10.17605/OSF.IO/MQKAJ.

## Ethics statement

The studies involving human participants were reviewed and approved by P-A-R-I-S Association Ethics Committee. The patients/participants provided their written informed consent to participate in this study.

## Author contributions

MB, DL, and JB: theoretical and conceptual design of the experiment. MB: data collection. BJ: data analysis. MB, BJ, DL, and JB: production of the manuscript. All authors contributed to the article and approved the submitted version.

## Footnotes

^1^“If two people are arguing ‘*if p will q*?' and are both in doubt as to *p*, (a) they are adding *p* hypothetically to their stock of knowledge and arguing on that basis about *q*; so that in a sense “*if p, q*” and “*if p*, $\bar{q}$” are contradictories. We can say (b) they are fixing their degrees of belief in *q given p*. (c) If *p* turns out false, these degrees of belief are rendered void. (d) If either party believes *p* for certain, the question ceases to mean anything to him except as a question about what follows from certain laws or hypotheses.” (Ramsey, 1990, p. 155, letter points added).

^2^Also known as *If-introduction* when it is a juxtaposition (Shao et al., 2022) and *Conjunctive Sufficiency* (Nute, 1980; Vidal and Baratgin, 2017), or *AND-to-IF* (Politzer and Baratgin, 2016), when it is a conjunction.

^3^“1 a été acceptée plus difficilement parce qu'on se trouve dans le domaine du vrai où ont cours certaines habitudes de cohérence que la phrase semble violer. Toutefois, cette exigence même de cohérence a amené quelques sujets à admettre la possibilité d'une relation entre termes appartenant à des domaines différents. Comme l'a dit l'un deux: ≪ Tout se tient. Pourquoi ne trouverait-on pas un jour une relation entre l'aritmétique et la couleurs des éléphants ?.” ≫ (1) was accepted with more difficulty because we are in the realm of truth where certain habits of coherence are being used that the statement seems to violate. However, this very requirement of coherence led some participants to admit the possibility of a relation between terms belonging to different domains. As one of them said: “Everything is linked. Why couldn't we find one day a relationship between arithmetic and elephant colors?”.” (Matalon, 1962, p. 89, our translation).

^4^Despite Kehler's label for this group, it should not be understood as requiring a strong causal relation but rather “could plausibly cause/result from” or, in some cases, “could plausibly be inferred from”.

^5^Calculated using the formula: *k*×*ln*(*n*)−2 × *ln*(*L*) where *k* is the number of parameters, *n* the number of samples, and *L* the likelihood.

^6^Calculated using the formula: 2*k*−2 × *ln*(*L*) where *k* is the number of parameters and *L* the likelihood.

^7^All models were tested using the lme4 R package (Bates et al., 2015), then compared using the R flexplot (Fife, 2021). The complete analysis is available at https://osf.io/ztcqf?view_only=a4a73b425cc245b482f5d64004342055. The full data repository is available at https://osf.io/mqkaj/?view_only=a4a73b425cc245b482f5d64004342055.

## References

[B1] Andraszewicz S. Scheibehenne B. Rieskamp J. Grasman R. Verhagen J. Wagenmakers E.-J. (2015). An introduction to Bayesian hypothesis testing for management research. J. Manage. 41, 521–543. 10.1177/0149206314560412

[B2] Asher N. Lascarides A. (2003). Logics of Conversation. Cambridge University Press.

[B3] Asher N. Vieu L. (2005). Subordinating and coordinating discourse relations. Lingua 115, 591–610. 10.1016/j.lingua.2003.09.017

[B4] Baratgin J. (2002). Is the human mind definitely not bayesian? A review of the various arguments. Curr. Psychol. Cogn. 21, 653–682.

[B5] Baratgin J. Douven I. Evans J. S. B. T. Oaksford M. Over D. E. Politzer G. (2015). The new paradigm and mental models. Trends Cogn. Sci. 19, 547–548. 10.1016/j.tics.2015.06.013 26412091

[B6] Baratgin J. Politzer G. (2006). Is the mind Bayesian? The case for agnosticism. Mind Soc. 5, 1–38. 10.1007/s11299-006-0007-1

[B7] Baratgin J. Politzer G. (2016). “Logic, probability and inference: a methodology for a new paradigm,” in Cognitive Unconscious and Human Rationality, eds L. Macchi, M. Bagassi, and R. Viale (Cambridge, MA: MIT Press), 119–142.

[B8] Bates D. Mächler M. Bolker B. Walker S. (2015). Fitting linear mixed-effects models using lme4. J. Stat. Softw. 67, 1–48. 10.18637/jss.v067.i01

[B9] Berto F. (2022). Williamson on indicatives and suppositional heuristics. Synthese 200, 1–12. 10.1007/s11229-022-03518-z 35194257 PMC8853152

[B10] Cruz N. (2022). Conceptual clarity and empirical testability: commentary on Knauff and Gazzo Castañeda (2022). Think. Reason. 10.1080/13546783.2022.2112757

[B11] Cruz N. Baratgin J. Oaksford M. Over D. E. (2015). Bayesian reasoning with IFS and ANDS and ORS. Front. Psychol. 6, 192. 10.3389/fpsyg.2015.00192 25762965 PMC4340177

[B12] Cruz N. Over D. Oaksford M. Baratgin J. (2016). “Centering and the meaning of conditionals,” in Proceedings of the 38th Annual Conference of the Cognitive Science Society, eds A. Papafragou, D. Grodner, D. Mirman, and J. C. Trueswell (Austin, TX), 1104–1109.32935375

[B13] Cruz N. Over D. E. (2023). “Independence conditionals,” in Conditionals: Logic, Linguistics, and Psychology, eds S. Kaufmann, D. E. Over, and G. Sharma (Palgrave Macmillan).

[B14] Douven I. (2008). The evidential support theory of conditionals. Synthese 164, 19–44. 10.1007/s11229-007-9214-5

[B15] Douven I. (2015). The Epistemology of Indicative Conditionals: Formal and Empirical Approaches. Cambridge University Press.

[B16] Douven I. (2017). How to account for the oddness of missing-link conditionals. Synthese 194, 1541–1554. 10.1007/s11229-015-0756-7

[B17] Douven I. (2022). Is the new paradigm a new paradigm? Comments on Knauff and Gazzo Castañeda. Think. Reason. 10.1080/13546783.2021.2017345

[B18] Douven I. Elqayam S. Mirabile P. (2022). Inference strength predicts the probability of conditionals better than conditional probability does. J. Memory Lang. 123, 104302. 10.1016/j.jml.2021.104302

[B19] Douven I. Elqayam S. Singmann H. van Wijnbergen-Huitink J. (2018). Conditionals and inferential connections: a hypothetical inferential theory. Cogn. Psychol. 101, 50–81. 10.1016/j.cogpsych.2017.09.002 29328949

[B20] Douven I. Verbrugge S. (2012). Indicatives, concessives, and evidential support. Think. Reason. 18, 480–499. 10.1080/13546783.2012.716009

[B21] Ducrot O. (2008). Dire et ne pas dire : Principes de sémantique linguistique, 3 Edn. Hermann, MO: Collection Savoir.

[B22] Edgington D. (1995). On conditionals. Mind 104, 235–329.

[B23] Elqayam S. (2017). “The new paradigm in psychology of reasoning,” in The Routledge International Handbook of Thinking and Reasoning, eds J. B. Linden and V. A. Thompson (Routledge), 130–150.

[B24] Elqayam S. Evans J. S. B. T. (2013). Rationality in the new paradigm: strict versus soft bayesian approaches. Think. Reason. 19, 453–470. 10.1080/13546783.2013.834268

[B25] Elqayam S. Over D. E. (2013). New paradigm psychology of reasoning: an introduction to the special issue edited by Elqayam, Bonnefon, and over. Think. Reason. 34, 249–265. 10.1080/13546783.2013.841591

[B26] Evans J. S. B. T. (2012). Questions and challenges for the new psychology of reasoning. Think. Reason. 18, 5–31. 10.1080/13546783.2011.637674

[B27] Evans J. S. B. T. Over D. E. (2004). If . Oxford: Oxford University Press.

[B28] Fife D. (2021). Flexplot: graphically-based data analysis. Psychol. Methods 27, 477–496. 10.1037/met0000424 34843276

[B29] Hobbs J. R. (1979). Coherence and coreference. Cogn. Sci. 3, 67–90.

[B30] Jeffreys H. (1961). The Theory of Probability, 3 Edn. Oxford University Press.

[B31] Johnson-Laird P. Khemlani S. S. Goodwin G. P. (2015). Logic, probability, and human reasoning. Trends Cogn. Sci. 19, 201–214. 10.1016/j.tics.2015.02.006 25770779

[B32] Johnson-Laird P. N. Khemlani S. (2022). What happened to the “new paradigm”? A commentary on knauff and gazzo castañeda (2022). Think. Reason. 10.1080/13546783.2021.2022532

[B33] Kehler A. (2002). Coherence, Reference, and the Theory of Grammar. Stanford, CA: CSLI Publications.

[B34] Kehler A. (2006). “Discourse coherence,” in The Handbook of Pragmatics, eds L. R. Horn and G. Ward (Blackwell Publishing), 241–265.

[B35] Knauff M. Estefania L. Castañeda G. (2021). When nomenclature matters: is the “new paradigm” really a new paradigm for the psychology of reasoning? *Think. Reason*. 10.1080/13546783.2021.1990126

[B36] Knott A. Dale R. (1994). Using linguistic phenomena to motivate a set of coherence relations. Discourse Process. 18, 35–62.

[B37] Krzyżanowska K. Collins P. J. Hahn U. (2017). Between a conditional's antecedent and its consequent: Discourse coherence vs. probabilistic relevance. Cognition 164, 199–205. 10.1016/j.cognition.2017.03.009 28453997

[B38] Krzyżanowska K. Collins P. J. Hahn U. (2021). True clauses and false connections. J. Memory Lang. 121, 104252. 10.1016/j.jml.2021.104252

[B39] Krzyżanowska K. Douven I. (2018). Missing-link conditionals: pragmatically infelicitous or semantically defective? Intercult. Pragmat. 15, 191–211. 10.1515/ip-2018-0004

[B40] Kuha J. (2004). AIC and BIC: comparisons of assumptions and performance. Sociol. Methods Res. 33, 188–229. 10.1177/0049124103262065

[B41] Lassiter D. (2022). Decomposing relevance in conditionals. Mind Lang. 1–25. 10.1111/mila.12418

[B42] Lassiter D. Baratgin J. (2021). Nested conditionals and genericity in the de finetti semantics. Thought J. Philos. 10, 42–52. 10.1002/tht3.478

[B43] Matalon B. (1962). “Étude génétique de l'implication,” in Implication, formalisation et logique naturelle, Vol. 16, eds E. W. Beth, J. B. Grize, R. Martin, B. Matalon, and J. Piaget (Presses Universitaires de France), 69–93.

[B44] Mirabile P. Douven I. (2020). Abductive conditionals as a test case for inferentialism. Cognition 200, 104232. 10.1016/j.cognition.2020.104232 32497915

[B45] Nute D. (1980). Topics in Conditional Logic, Vol. 20. Dordrecht: Springer.

[B46] Oaksford M. (2021). Mental models, computational explanation and bayesian cognitive science: commentary on Knauff and Gazzo Castañeda (2022). Think. Reason. 10.1080/13546783.2021.2022531

[B47] Oaksford M. Chater N. (2001). The probabilistic approach to human reasoning. Trends Cogn. Sci. 5, 349–357. 10.1016/S1364-6613(00)01699-5 11477004

[B48] Oaksford M. Chater N. (2007). Bayesian Rationality: The Probabilistic Approach to Human Reasoning. Oxford: Oxford University Press. 10.1017/S0140525X09000284 19210833

[B49] Oaksford M. Chater N. (2013). Dynamic inference and everyday conditional reasoning in the new paradigm. Think. Reason. 19, 346–379. 10.1080/13546783.2013.80816330024177

[B50] Oaksford M. Chater N. (2020). “Integrating causal bayes nets and inferentialism in conditional inference,” in Logic and Uncertainty in the Human Mind: A Tribute to David E. Over, eds S. Elqayam, I. Douven, I., J. S. B. T. Evans, and N. Cruz (Routledge), 116–132.

[B51] Over D. E. (2009). New paradigm psychology of reasoning. Think. Reason. 15, 431–438. 10.1080/13546780903266188

[B52] Over D. E. (2020). “The development of the new paradigm in the psychology of reasonings,” in Logic and Uncertainty in the Human Mind: A Tribute to David E. Over, eds S. Elqayam, I. Douven, I., J. S. B. T. Evans, and N. Cruz (Routledge), 161–177.

[B53] Over D. E. (2021). The new paradigm and massive modalization. Think. Reason. 1–7. 10.1080/13546783.2021.201734631774734

[B54] Over D. E. Cruz N. (2023). “Indicative and counterfactual conditionals in the psychology of reasoning,” in Conditionals: Logic, Linguistics, and Psychology, eds S. Kaufmann, D. E. Over, and G. Sharma (Palgrave Macmillan).

[B55] Politzer G. Baratgin J. (2016). Deductive schemas with uncertain premises using qualitative probability expressions. Think. Reason. 22, 78–98. 10.1080/13546783.2015.1052561

[B56] Raftery A. E. (1999). Bayes factors and BIC: comment on “a critique of the Bayesian information criterion for model selection”. Sociol. Methods Res. 27, 411–427. 10.1177/0049124199027003005

[B57] Ramsey F. P. (1990). “General propositions and causality,” in F. P. Ramsey: Philosophical Papers, ed D. H. Mellor (Cambridge: Cambridge University Press), 145–163.

[B58] Rostworowski W. Pietrulewicz N. Bedkowski M. (2021). Conditionals and specific links–an experimental study. Synthese 199, 7365–7399. 10.1007/s11229-021-03119-2

[B59] Shao J. Tikiri Banda D. Baratgin J. (2022). A study on the sufficient conditional and the necessary conditional with chinese and french participants. Front. Psychol. 13, 787588. 10.3389/fpsyg.2022.787588 35282197 PMC8907880

[B60] Sikorski M. (2022). The Ramsey test and evidential support theory. J. Logic Lang. Inform. 31, 493–504. 10.1007/s10849-022-09364-z

[B61] Skovgaard-Olsen N. Collins P. Krzyżanowska K. Hahn U. Klauer K. C. (2019). Cancellation, negation, and rejection. Cogn. Psychol. 108, 42–71. 10.1016/j.cogpsych.2018.11.002 30593995

[B62] Skovgaard-Olsen N. Singmann H. Klauer K. C. (2016). The relevance effect and conditionals. Cognition 150, 26–36. 10.1016/j.cognition.2015.12.017 26848733

[B63] Skovgaard-Olsen N. Singmann H. Klauer K. C. (2017). Relevance and reason relations. Cogn. Sci. 41, 1202–1215. 10.1111/cogs.12462 28032658

[B64] Stalnaker R. C. (1970). Probability and conditionals. Philos. Sci. 37, 64–80.

[B65] Txurruka I. G. (2003). The natural language conjunction *and*. Linguist. Philos. 26, 255–285. 10.1023/A:1024117423963

[B66] Vidal M. Baratgin J. (2017). A psychological study of unconnected conditionals. J. Cogn. Psychol. 29, 769–781. 10.1080/20445911.2017.1305388

[B67] Wang M. Over D. Liang L. (2022). What is required for the truth of a general conditional? Q. J. Exp. Psychol. 75, 2105–2117. 10.1177/17470218221089331 35262439

[B68] Wolf F. Gibson E. Desmet T. (2004). Discourse coherence and pronoun resolution. Lang. Cogn. Process. 19, 665–675. 10.1080/01690960444000034

