# Supplementary material for: Coherence, not conditional meaning, accounts for the relevance effect
Source: Front Psychol. 2023 May 15;14:1150550. doi: 10.3389/fpsyg.2023.1150550 (PMC10225734; doi:10.3389/fpsyg.2023.1150550)
Supplement: Supplementary file 1 [file Data_Sheet_1.PDF]

Supplementary Material

1 STATEMENTS USED DURING THE EXPERIMENT

Table S1. All 24 items in french

Statement form	Item
	Inferential link, Intended relation of result
Conditional	Si Lucie fait la taille requise, [par conséquent/malgré cela] elle pourra monter dans le manège.
Conjunction	Lucie fait la taille requise et [par conséquent/malgré cela] elle pourra monter dans le manège.
Juxtaposition	Lucie fait la taille requise. [Par conséquent/Malgré cela] elle pourra monter dans le manège.
Conditional	Si tu as aimé le roman 1984, [par conséquent/malgré cela] tu devrais aimer le roman Le meilleur des mondes.
Conjunction	Tu as aimé le roman 1984 et [par conséquent/malgré cela] tu devrais aimer le roman Le meilleur des mondes.
Juxtaposition	Tu as aimé le roman 1984. [Par conséquent/Malgré cela] tu devrais aimer le roman Le meilleur des mondes.
	Inferential link, Intended relation of explanation
Conditional	Si le vase est casé, [par conséquent/malgré cela] quelqu'un l'a fait tomber.
Conjunction	Le vase est casé et [par conséquent/malgré cela] quelqu'un l'a fait tomber.
Juxtaposition	Le vase est casé. [Par conséquent/Malgré cela] quelqu'un l'a fait tomber.
Conditional	Si les bourgeons s'ouvrent cette semaine, [par conséquent/malgré cela] nous sommes au printemps.
Conjunction	Les bourgeons s'ouvrent cette semaine et [par conséquent/malgré cela] nous sommes au printemps.
Juxtaposition	Les bourgeons s'ouvrent cette semaine. [Par conséquent/Malgré cela] nous sommes au printemps.
	No inferential link, intended relation of violated expectation
Conditional	Si John a dit que Bill était sympa, [par conséquent/malgré cela] Bill l'a frappé.
Conjunction	John a dit que Bill était sympa et [par conséquent/malgré cela] Bill l'a frappé.
Juxtaposition	John a dit que Bill était sympa. [Par conséquent/Malgré cela] Bill l'a frappé.
Conditional	Si Marie est partie très vite de la soirée, [par conséquent/malgré cela] l'hôte était très reconnaissant envers elle quand elle est partie.
Conjunction	Marie est partie très vite de la soirée et [par conséquent/malgré cela] l'hôte était très reconnaissant envers elle quand elle est partie.
Juxtaposition	Marie est partie très vite de la soirée. [Par conséquent/Malgré cela] l'hôte était très reconnaissant envers elle quand elle est partie.
	No inferential link, Intended relation of denial of preventer
Conditional	Si Martin a tout fait pour arriver à l'heure, [par conséquent/malgré cela] son bus part sans lui.
Conjunction	Martin a tout fait pour arriver à l'heure et [par conséquent/malgré cela] son bus part sans lui.
Juxtaposition	Martin a tout fait pour arriver à l'heure. [Par conséquent/Malgré cela] son bus part sans lui.
Conditional	Si l'équipe de France a tout fait pour revenir au score, [par conséquent/malgré cela] elle a perdu le match.
Conjunction	L'équipe de France a tout fait pour revenir au score et [par conséquent/malgré cela] elle a perdu le match.
Juxtaposition	L'équipe de France a tout fait pour revenir au score. [Par conséquent/Malgré cela] elle a perdu le match.

Table S2. *All 24 Items in English*

Statement form	Item
	Inferential link, Intended relation of result
Conditional	If Lucie is tall enough,[consequently/in spite of that] she will be able to ride the carousel.
Conjunction	Lucy is tall enough and [consequently/in spite of that] will be able to ride the carousel.
Juxtaposition	Lucy is tall enough.[Consequently/In spite of that] she will be able to ride the carousel.
Conditional	If you liked the novel 1984, [consequently/in spite of that] you should like Brave New World.
Conjunction	You liked 1984 and [consequently/in spite of that] you should like Brave New World.
Juxtaposition	You liked the novel 1984. [Consequently/In spite of that] you should like Brave New World.
	Inferential link, Intended relation of explanation
Conditional	If the vase is broken, [consequently/in spite of that] someone dropped it.
Conjonction	The vase is broken and [consequently/in spite of that] someone dropped it.
Juxtaposition	The vase is broken. [Consequently/In spite of that] someone dropped it.
Conditional	If the buds open this week, [consequently/in spite of that] it's spring.
Conjunction	The buds open this week and [consequently/in spite of that] it is spring.
Juxtaposition	The buds open this week. [Consequently/In spite of that] we are in spring.
	No inferential link, Intended relation of violated expectation
Conditional	If John said Bill was nice, [consequently/in spite of that] Bill hit him.
Conjunction	John said Bill was nice and [consequently/in spite of that] Bill hit him.
Juxtaposition	John said Bill was nice. [Consequently/In spite of that] Bill hit him.
Conditional	If Mary left the party in a hurry, [consequently/in spite of that] the host was very grateful to her when she left.
Conjunction	Mary left the party in a hurry and [consequently/in spite of that] the host was very grateful to her when she left.
Juxtaposition	Mary left the party in a hurry. [Consequently/In spite of that] the host was very grateful to her when she left.
	No inferential link, Intended relation of denial of preventer
Conditional	If Martin did everything possible to arrive on time, [consequently/in spite of that] his bus leaves without him.
Conjunction	Martin did everything he could to arrive on time and [consequently/in spite of that] his bus leaves without him.
Juxtaposition	Martin did everything he could to be on time. [Consequently/In spite of that] his bus leaves without him.
Conditional	If the French team did everything to come back to the score, [consequently/in spite of that] they lost the game.
Conjunction	The French team did everything to come back to the score and [consequently/in spite of that] they lost the game.
Juxtaposition	The French team did everything to come back to the score. [Consequently/In spite of that] they lost the game.

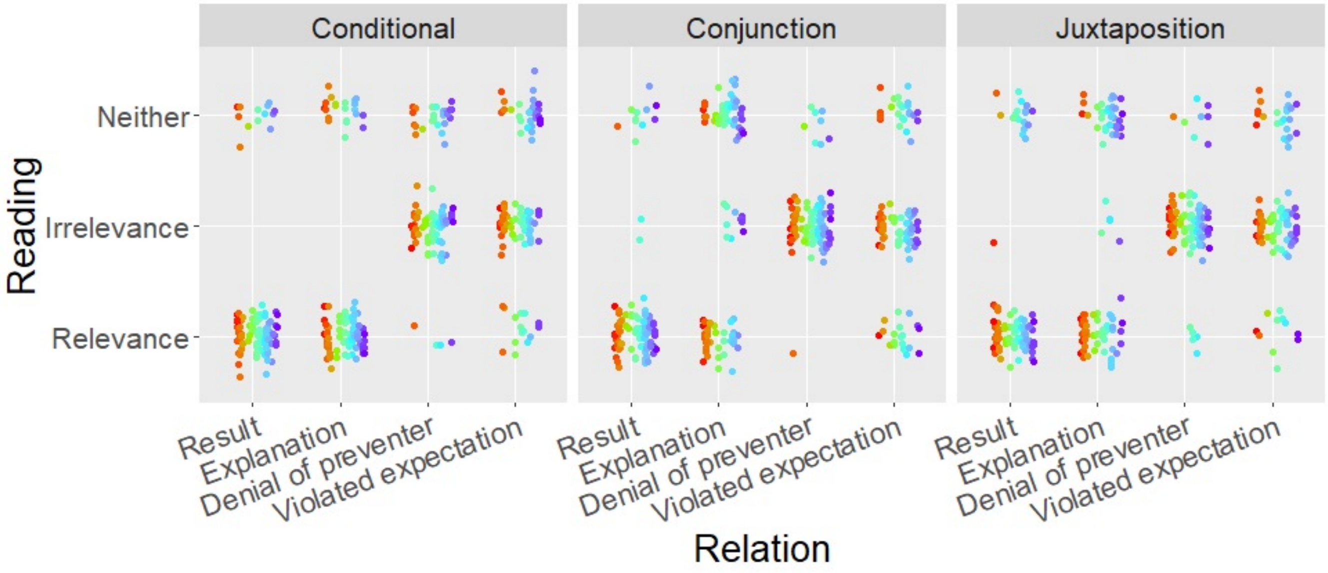

Each dot corresponds to an answer, and each of the 50 colors (on the gradient) corresponds to a single participant.

Figure S1. Graphical representation of all the data gathered (N=1200, Participants=50).
